# A nationwide cross-sectional study on the association of patient-level factors with financial anxiety in the context of chronic medical conditions

**DOI:** 10.1038/s41598-023-36282-2

**Published:** 2023-06-26

**Authors:** Abbas M. Hassan, Carrie K. Chu, Jun Liu, Rebekah Angove, Gabrielle Rocque, Kathleen D. Gallagher, Adeyiza O. Momoh, Nicole E. Caston, Courtney P. Williams, Stephanie Wheeler, Anaeze C. Offodile

**Affiliations:** 1 Department of Plastic and Reconstructive Surgery, The University of Texas MD Anderson Cancer Center, 1400 Pressler St., Unit 1488, Houston, TX 77030 USA; 2 Patient Advocate Foundation, Hampton, VA USA; 3 Division of Hematology and Oncology, Department of Medicine, University of Alabama at Birmingham, Birmingham, AL USA; 4 Section of Plastic Surgery, Department of Surgery, University of Michigan, Ann Arbor, MI USA; 5 Department of Health Policy and Management, Gillings School of Global Public Health, The University of North Carolina at Chapel Hill, Chapel Hill, NC USA; 6 Department of Health Policy Services Research, The University of Texas MD Anderson Cancer Center, Houston, TX USA; 7 Baker Institute for Public Policy, Rice University, Houston, TX USA

**Keywords:** Health policy, Health services, Quality of life

## Abstract

Patient-level characteristics associated with the prevalence and severity of financial anxiety have yet to be described. We conducted a cross-sectional analysis of survey data assessing financial anxiety in patients with chronic medical conditions in December 2020. 1771 patients (42.6% response rate) participated in the survey. Younger age (19–35 age compared to ≥ 75 age) (β, 5.86; 95% CI 2.10–9.63), male sex (β, − 1.9; 95% CI − 3.1 to − 0.73), Hispanic/Latino race/ethnicity (compared with White patients) (β, 2.55; 95% CI 0.39–4.71), household size ≥ 4 (compare with single household) (β, 4.54; 95% CI 2.44–6.64), household income of ≥ $96,000-$119,999 (compared with ≤ $23,999) (β, − 3.2; 95% CI − 6.3 to 0.04), single marital status (compared with married) (β, 2.18; 95% CI 0.65–3.71), unemployment (β, 2.07; 95% CI 0.39–3.74), high-school education (compared with advanced degrees) (β, 3.10; 95% CI 1.32–4.89), lack of insurance coverage (compared with private insurance) (β, 6.05; 95% CI 2.66–9.45), more comorbidities (≥ 3 comorbidities compared to none) (β, 2.95; 95% CI 1.00–4.90) were all independently associated with financial anxiety. Patients who are young, female, unmarried, and representing vulnerable sub-populations are at elevated risk for financial anxiety.

## Introduction

High out-of-pocket spending attributable to the receipt of medical care is an increasingly critical issue for patients in the United States (US). In 2018, nearly 140 million adults reported difficulty with paying for medical expenses in the US^1^. Chronic medical conditions are among the leading causes of death and disability in the United States, and as a result, they account for a disproportionate amount of health-care utilization and spending^2,3^. Six in ten individuals in the US have at least one chronic medical condition, with annual direct costs estimated at $1. trillion^4^. Furthermore, the average annual direct health care expenditure for a patient with a chronic medical condition is $6,032, which is nearly 5 times that of a person without a chronic medical condition^5^.

Financial anxiety which is a distinct construct from financial distress/hardship, denotes the affective experience of financial distress and encompasses a wide range of anxiety symptoms and pervasive concerns about one’s financial situation, which are driven by a perception that existing and future economic resources are insufficient to meet basic needs^6,7^. Financial anxiety can be conceptualized as a psychosocial syndrome that has significant implications for financial behavior and although interrelated, it is a distinct construct from general anxiety and depression^7^. The projected trends in the burden of several chronic conditions, increased utilization of patient cost-sharing (i.e., high deductible health plans), and escalating health care costs has catalyzed concerns about an increased prevalence of financial anxiety in near- and long-term^1,8^. These findings are alarming because financial anxiety is associated with negative impacts on patients’ financial, physical, and emotional well-being, as well as their adherence to treatment recommendations^9,10^. Patients experiencing financial distress are also more likely to report a higher symptom burden, lower quality of life, increased risk of death, as well as forgo physician visits, mental health care and other services when compared to patients who report not experiencing financial distress^11–13^.

We posit that the relationship between patients’ perceptions and their emotional reactions to their financial difficulty may be differentially driven by socioeconomic factors. For instance, patients with comparable income levels may experience varying degrees of financial anxiety depending on their consumption priorities and spending tendencies related to housing, transportation, healthcare, and food^14,15^. However, to the best of our knowledge, no study has yet examined the association of socioeconomic factors with financial anxiety among patients with chronic medical conditions.

Our goal is to leverage a cross-sectional national survey to investigate the determinants of financial anxiety in patients with chronic medical conditions, and determine if an active cancer diagnosis is associated with higher financial anxiety. This is salient because elucidating the relationship of socioeconomic factors with financial anxiety can help providers and health services researchers identify strategies to improve care delivery, health resource utilization, and quality-of-life outcomes. We hypothesized that certain chronic conditions, specifically a history of cancer and certain socioeconomic characteristics such as race and ethnicity, insurance status, and income level are differentially associated with financial anxiety.

## Results

### Patient demographics

A total of 1771 out of 4149 participants responded to our survey (42.6% response rate) and comprised our analytic sample. We observed that 57% (n = 1013) of respondents were female, and 63% (n = 1116) were White. Most patients (57.7%, n = 1021) were between the ages of 56 and 75. Complete patient demographics are outlined in Table 1. Thirty-five percent (n = 627) of patients reported an active cancer diagnosis. The highest level of educational attainment was some college (36.6%, n = 648), and the annual household income range for most patients was $24,000–$47,999 (42.9%, n = 759). Medicare was the most common form of insurance for patients (57.7%), followed by private insurance (19.1%). The mean (± SD) FAS score was 24.4 ± 13.1, and the median (IQR) was 23 (12–35).

**Table 1:** Respondent demographic and clinical characteristics (N = 1771).

Patient characteristic	N (%)
Age group (years)	82 (4.6)
19 to 35	583 (32.9)
36 to 55	1021 (57.7)
56 to 75	81 (4.6)
Sex	
Female	1013 (57.2)
Male	748 (42.2)
Race and ethnicity	
White	1116 (63)
Black	350 (19.8)
Hispanic/Latino	114 (6.4)
Other	120 (6.8)
Unknown	71 (4)
Annual household income	
≤ $23,999	573 (32.4)
$24,000–$47,999	759 (42.9)
$48,000–$71,999	282 (15.9)
$72,000–$95,999	75 (4.2)
$96,000–$119,999	62 (3.5)
Unknown	20 (1.1)
Household size	
1	660 (37.3)
2	576 (32.5)
3	227 (12.8)
4 +	308 (17.4)
Marital status	
Married, or living as married	664 (37.5)
Divorced/separated/widow	420 (23.7)
Single	642 (36.3)
Unknown	45 (2.5)
Employment status	
Employed	943 (53.2)
Disabled	5 (0.3)
Retired	580 (32.7)
Unemployed/other	243 (13.7)
Education level	
Advanced degree	319 (18)
Bachelor’s degree	474 (26.8)
High school or less	326 (18.4)
Some college	648 (36.6)
Unknown	4 (0.2)
Insurance	
Medicare	1022 (57.7)
ACA Exchange	93 (5.3)
COBRA	28 (1.6)
Medicaid	142 (8)
None	53 (3)
Private employer	95 (5.4)
Unknown	338 (19.1)
Region	
Midwest	285 (16.1)
Northeast	252 (14.2)
South	908 (51.3)
West	316 (17.8)
Unknown	10 (0.6)
Rural–urban commuting area	
Rural	353 (19.9)
Suburban	821 (46.4)
Urban	541 (30.5)
Unknown	56 (3.2)
Number of comorbidities	
0	239 (13.5)
1–2	749 (42.3)
3 +	725 (40.9)
Unknown	58 (3.3)
Telemedicine utilization	
None	502 (28.3)
Low volume utilizers (1 visit)	247 (13.9)
Modest volume utilizers (2–5 visits)	742 (41.9)
High volume utilizers (> 5 visits)	272 (15.4)
Active cancer diagnosis	627 (35.4)
Financial anxiety scale	
Mean ± SD	24.4 ± 13.1
Median (IQR)	23 (12–35)

*ACA* affordable care act, *COBRA* consolidated omnibus budget reconciliation act, *S.D* standard deviation, *IQR* interquartile range.

### Factors associated with financial anxiety (Table 2)

**Table 2:** Univariable and multivariable linear regression models for the impact of socioeconomic factors on financial anxiety (N = 1771).

Patient characteristic	Univariable analysis		Multivariable analysis	
	β (95% CI)	*P* value	β (95% CI)	*P* value
Age group (years)				
Over 75	0.38 (− 2.47 to 3.22)	0.795	2.47 (− 0.30 to 5.24)	0.081
56 to 75	Ref		Ref	
36 to 55	− 8.19 (− 9.44 to − 6.94)	**< 0.001**	− 2.34 (− 3.96 to − 0.72)	**0.005**
19 to 35	− 14.5 (− 17.35 to − 11.62)	**< 0.001**	− 4.99 (− 8.11− − 1.86)	**0.002**
Sex				
Female	Ref		Ref	
Male	− 6.5 (− 7.7 to − 5.3)	**< 0.001**	− 1.9 (− 3.1 to − .73)	**0.002**
Race and ethnicity				
White	Ref		Ref	
Black	4.22 (2.67–5.77)	**< 0.001**	0.08 (− 1.4 to 1.51)	0.913
Hispanic/Latino	4.86 (2.37–7.35)	**< 0.001**	2.55 (0.39–4.71)	**0.021**
Other	3.51 (1.08–5.94)	**0.005**	2.40 (0.26–4.54)	**0.028**
Annual household income				
≤ $23,999	Ref		Ref	
$24,000–$47,999	− 3.4 (− 4.8 to − 2.0)	**< 0.001**	− 1.2 (− 2.4 to 0.11)	0.074
$48,000–$71,999	− 5.9 (− 7.7 to − 4.1)	**< 0.001**	− 3.0 (− 4.8 to − 1.2)	**0.001**
$72,000–$95,999	− 1.3 (− 4.4 to 1.82)	0.419	− 2.2 (− 5.0 to 0.73)	0.144
$96,000–$119,999	− 1.6 (− 5.0 to 1.76)	0.349	− 3.2 (− 6.3 to 0.04)	0.053
Household size				
1	Ref		Ref	
2	− .59 (− 2.0 to 0.80)	0.406	1.59 (0.04–3.15)	**0.045**
3	7.67 (5.79–9.55)	**< 0.001**	4.96 (2.96–6.96)	**< 0.001**
4 +	8.53 (6.84–10.2)	**< 0.001**	4.54 (2.44–6.64)	**< 0.001**
Marital status				
Married, or living as married	Ref		Ref	
Divorced/separated/widow	4.10 (2.52–5.68)	**< 0.001**	3.52 (1.94–5.10)	**< 0.001**
Single	2.54 (1.13–3.95)	**< 0.001**	2.18 (0.65–3.71)	**0.005**
Employment status				
Employed	Ref		Ref	
Disabled	− 5.4 ( − 16 to 5.11)	0.316	− 5.9 ( − 16 to 4.04)	0.245
Retired	− 9.8 ( − 11 to − 8.6)	**< 0.001**	− 5.9 (− 7.3 to − 4.5)	**< 0.001**
Unemployed/other	4.84 (3.16–6.52)	**< 0.001**	2.07 (0.39–3.74)	**0.016**
Region				
Midwest	Ref		Ref	
Northeast	0.50 (− 1.7 to 2.71)	0.660	1.19 (− .69 to 3.08)	0.215
South	1.19 (− .55 to 2.93)	0.178	1.21 (− .28 to 2.71)	0.112
West	− .92 (− 3.0 to 1.17)	0.386	0.30 (− 1.5 to 2.12)	0.748
Education level				
Advanced degree	Ref		Ref	
Bachelor’s degree	2.21 (0.37–4.05)	**0.018**	1.43 (− .15 to 3.00)	**0.076**
High school or less	3.68 (1.94–5.42)	**< 0.001**	1.52 (− .02 to 3.06)	**0.054**
Some college	5.48 (3.48–7.48)	**< 0.001**	3.10 (1.32–4.89)	**0.001**
Insurance				
Medicare	Ref		Ref	
ACA exchange	− 3.0 (− 4.5 to − 1.5)	**< 0.001**	1.45 (− .19 to 3.10)	0.083
COBRA	1.67 (− 1.2 to 4.53)	0.251	2.49 (− .12 to 5.09)	0.061
Medicaid	4.82 (0.02–9.62)	**0.049**	4.67 (0.37–8.97)	**0.033**
None	8.97 (6.53–11.4)	**< 0.001**	4.73 (2.37–7.08)	**< 0.001**
Other/unknown	10.1 (6.53–13.7)	**< 0.001**	6.05 (2.66–9.45)	**< 0.001**
Private employer	3.01 (0.18–5.84)	**0.037**	2.98 (0.39–5.56)	**0.024**
Rural–urban commuting area				
Rural	Ref		Ref	
Suburban	− 1.5 (− 3.1 to 0.12)	0.069	0.04 (− 1.4 to 1.43)	0.954
Urban	− .01 (− 1.8 to 1.74)	0.989	0.91 (− .62 to 2.45)	0.243
Active cancer diagnosis				
None	Ref		Ref	
Yes	1.35 (0.08 to 2.62)	**0.037**	0.99 (− .34 to 2.31)	0.144
Disease type				
Cardiopulmonary	Ref		Ref	
Cancer	6.21 (2.91–9.52)	**< 0.001**	2.00 (− 0.99 to 4.99)	0.190
Autoimmune	3.19 (− 0.13 to 6.52)	0.060	1.74 (− 1.28 to 4.75)	0.259
Genetic disorders	− 4.56 (− 10.34 to 19.46)	0.549	1.96 ( − 11.30 to 15.21)	0.772
Neurologic	11.56 (3.66 to 19.45)	**0.004**	8.61 (1.63 to 15.60)	**0.016**
Inflammatory diseases	5.87 (2.10–9.67)	**0.003**	1.47 (− 1.96 to 4.90)	0.400
Others	2.99 (− 0.90 to 6.87)	0.132	− 0.41 (− 3.95− 3.14)	0.821
Multiple	7.94 (4.78 to 11.09)	**< 0.001**	3.71 (0.86–6.56)	**0.011**

*β* beta coefficients, *CI* confidence interval, *ACA* affordable care act, *COBRA* consolidated omnibus budget reconciliation act. Significant values are in bold.

In adjusted models, patients in the 56–75 and over 75 age groups had significantly lower FAS scores compared to patients in the 36–55 years group (β, − 2.34; 95% CI − 3.96 to − 0.72 and β, − 4.99; 95% CI − 8.11 to 1.86, respectively). In contrast, there was no significant difference in FAS scores observed between the 19–35 age group and the 36–55 years age group (β, 2.47; 95% CI − 0.30 to 5.24). Male patients (β, − 1.9; 95% CI − 3.1 to − 0.73) had significantly lower financial anxiety scores than female patients. Compared with White patients, Hispanic/Latino patients (β, 2.55; 95% CI 0.39–4.71) had higher financial anxiety scores. Increasing household size was significantly associated with higher financial anxiety scores relative to a single household. Similarly, increasing household income was associated with lower financial anxiety scores.

Single (β, 2.18; 95% CI 0.65–3.71), and divorced/separated/widowed (β, 3.52; 95% CI 1.94–5.10) patients had significantly higher financial anxiety scores than married patients. Compared with employed patients, those who were retired had lower financial anxiety scores (β, − 5.9; 95% CI − 7.3 to − 4.5), while unemployed patients had higher scores (β, 2.07; 95% CI 0.39–3.74). Compared with patients with advanced degrees, those with a high school diploma or less had higher financial anxiety scores (β, 3.10; 95% CI 1.32–4.89). Patients insured by COBRA health plans (β, 4.67; 95% CI 0.37–8.97), Medicaid (β, 4.73; 95% CI 2.37–7.08), and the non-insured (β, 6.05; 95% CI 2.66–9.45) had higher financial anxiety scores compared with those with private insurance. Compared with patients with no comorbid conditions, those with ≥ 3 comorbidities had higher financial anxiety scores (β, 2.95; 95% CI 1.00–4.90). Lastly, an active cancer diagnosis was not associated with higher financial anxiety scores (β, 0.99; 95% CI − 0.34 to 2.31).

### Disease type and financial anxiety

We conducted a subgroup analysis examining the association of disease type and financial anxiety (Table 3). We found a significant difference in the mean and median FAS scores according to disease type (*p* < 0.001). Patients with neurological disease had the highest mean FAS scores (30 ± 13.12), followed by multiple disease (26.38 ± 13.34), cancer (24.66 ± 12.9), and inflammatory disease (24.31 ± 13.38). Conversely, patients with cardiopulmonary disease (18.44 ± 12.26) and immune conditions (21.64 ± 11.98) had the lowest FAS scores. In multivariable regression (Table 2), patients with neurologic disease (β, 8.61; 95% CI 1.63–15.60), and multiple conditions (β, 3.71; 95% CI 0.86–6.56) had significantly higher FAS scores compared to patients with cardiopulmonary conditions.

**Table 3:** Financial anxiety according to disease type.

	Financial anxiety scale		*P* value
	Mean + SD	Median (IQR)	
*Disease type*			** < 0.001**
Cancer	24.66 ± 12.9	24 (13, 35)	
Cardiopulmonary	18.44 ± 12.26	13.5 (8, 27)	
Immune conditions	21.64 ± 11.98	19 (10.5, 30)	
Genetic disorders	23 ± 15.52	22 (8, 39)	
Neurologic	30 ± 13.12	32.5 (21, 39)	
Inflammatory diseases	24.31 ± 13.38	22 (12, 33)	
Others	21.43 ± 11.9	19 (11, 30)	
Multiple	26.38 ± 13.34	26 (14, 37)	

*SD* standard deviation, *IQR* interquartile range. Significant values are in bold.

## Discussion

In this cross-sectional study of patients with chronic medical conditions who are receiving social needs and financial assistance, we sought to illuminate the association of patient-level characteristics (i.e., demographic and socioeconomic), and an active cancer diagnosis with financial anxiety. In adjusted models, the following factors were identified as having an independent association with worsening financial anxiety: younger age, Hispanic/Latino ethnicity, female sex, larger household size, lower household income, single marital status, unemployment, lower education levels, high comorbidity burden, lack of insurance coverage, COBRA beneficiaries, and Medicaid insurance. Active cancer diagnosis was not found to be associated with an increased risk of financial anxiety. These findings are salient because financial anxiety has been associated with higher level of psychological distress, which can have deleterious effects on medical decision-making, treatment or screening adherence, and lead to significant adverse health outcomes, and higher risk of mortality^16–21^.

Previous studies have shown that socioeconomic factors have a moderating effect that could worsen financial distress^22–24^. In the current study, we found that patients with lower socioeconomic status (i.e., income-level, educational attainment, employment status and insurance type) demonstrated a more severe manifestation of financial anxiety. Plausible explanations for our findings is that relative to patients with socio-economic advantages, those with lower socioeconomic status (1) have lower engagement in health-promoting habits and lower access to healthcare^25,26^; (2) are less aware of and prioritize mental health^27,28^; (3) do not have access to adequate economic, social, and psychological resources to alleviate financial stress and psychological distress^29,30^; and (4) have fewer coping mechanisms when faced with adversity^31,32^. The confluence of these factors might translate to a higher prevalence of depression, anxiety, psychosomatic symptoms, lower subjective well-being and reduced self-esteem^33,35^. Limited access to physical and mental health care can also result in greater mental health deterioration^25^. 

Our noted associations of financial anxiety with race and ethnicity, sex, marital status, and age are concordant with previous studies^33–35^. Married individuals have been documented as having improved emotional, financial, and psychological well-being, including reducing the risk of psychological distress, depression, and psychiatric illnesses^36,37^. This is likely because such individuals may have, via their partners, immediate practical, emotional, and financial assistance^38^. Grable et al.^39^ found that racial and ethnic minorities reported higher levels of financial stress than White individuals. In a previous study, younger age was also found to correlate with higher financial distress, most likely due to competing financial pressures (e.g. student loans, car payments), limited opportunities to accrue savings, and maladaptive financial behaviors^40,41^. Consistent with previous findings, we found that women appeared to be particularly vulnerable to financial distress, implying that more research is needed to better understand sex-based disparities in financial anxiety and mental health^33–35^.

There is increasing appreciation that financial anxiety may be especially severe in patients with pre-existing medical conditions^12,31,42^. In a cross-sectional study using data from the 2016 National Health Interview Survey (NHIS), investigators found a greater burden of financial worries among US adults with at least one or more chronic health conditions compared to those adults without chronic health conditions^24^. Consistent with these findings, our study demonstrated that patients with ≥ 3 comorbidities had significantly higher financial anxiety compare with those without comorbid conditions. Furthermore, patients with chronic medical conditions have higher out-of-pocket costs (5–10 times higher than those without a chronic condition) and face a disproportionate financial burden as a result of their complex healthcare needs^43–45^. Lastly, we report no significant relationship between an active cancer diagnosis and financial anxiety, despite the high likelihood that recipients of PAF services satisfy criterion for financial toxicity. This finding suggests that financial anxiety and financial toxicity are underpinned by different mechanisms of action and highlight the need for conceptual frameworks that deepen our understanding of financial anxiety.

The significant association between FAS scores and disease type revealed in our study is important because it highlights the unique financial challenges faced by patients with various chronic medical conditions. The identification of disease-specific differences in FAS scores suggests that financial anxiety is not a one-size-fits-all problem, and that interventions and support services should be tailored to the specific needs of patients with different disease types. Our results suggest that patients with certain conditions, such as neurological disease and those with multiple conditions, may be at increased risk for financial anxiety and may require additional financial counseling and assistance to manage their healthcare costs. These findings demonstrate the importance of considering disease-specific characteristics when addressing financial anxiety in the context of chronic medical conditions and highlight the need for the design of more targeted and personalized approaches to healthcare delivery and financial counseling.

Our results hold several implications for medical providers, mental health professionals, and policymakers. Medical providers must be aware that some patient populations may be more susceptible to the negative consequences of financial anxiety and have unmet health care needs, particularly the uninsured, unemployed, low-income households, and those with a high comorbidity burden. The data presented in this study could serve as a catalyst for health care delivery systems to proactively identify adults at risk of high financial anxiety, conduct regular screening and monitoring, and facilitate timely referrals to financial anxiety mitigation counseling and therapy services. Policy-level interventions should also be implemented to provide adequate resources and attention to individuals who are especially vulnerable to financial anxiety, such as by facilitating referrals to financial education, navigation programs, and counseling services, so that individuals can learn and develop coping strategies to manage their financial anxiety. Lastly, we hope that our results will incite interest in the research community to deepen our understanding of the impact of financial anxiety on condition-specific quality of life, patterns of acute care utilization, and overall survival.

Our study has certain limitations that warrant mention, including a cross-sectional, observational design that could lead to unmeasured confounding and inability to establish causality. Our study evaluated an underserved and limited-resource patient population, so it might not be representative of the overall general population. Moreover, our study included a nationwide cohort of PAF service recipients who may already be experiencing financial concerns; therefore, our findings may not be generalizable to all patients with chronic medical conditions. Our reported data may be biased toward those who are able to use telephonic and online non-profit services and access English language web-based surveys. We only included patients in our study who were able to receive survey communications from PAF via email. Individuals who did not fulfill this criterion were not included in the study, which might have resulted in selection bias. Additionally, the self-reported survey data used in this study can introduce recall bias and potentially imprecision. Finally, we were unable to conduct a non-responder analysis due to our sampling frame, so we are unable to identify any significant systematic differences between those who took part in our study and those who did not.

## Conclusion

This study evaluated the association between socioeconomic factors and financial anxiety among a nationwide cohort of patients with chronic medical conditions. Our results indicate that patients who are young, female, unmarried and representing vulnerable sub-populations (i.e., minority groups, uninsured, unemployed, low educational attainment, and low-income levels) are at elevated risk for financial anxiety. An active cancer diagnosis was not associated with higher financial anxiety compared with other chronic medical conditions. Policy-level interventions should be implemented to provide adequate resources and attention to individuals who are especially vulnerable to financial anxiety.

## Methods

### Study design

This is a cross-sectional analysis of patients with chronic medical conditions who responded to a nationwide survey distributed by The Patient Advocate Foundation (PAF). PAF is a 501(c)(3) non-profit organization that provides social needs navigation and various forms of financial assistance to patients with a diagnosis of a chronic illness within the US. A detailed overview of the survey methodology was previously published^46^. In brief, the survey was electronically distributed, via email, from December 2 -23, 2020. Participation was completely voluntary and non-response generated up to three emails reminders. Random drawings were held to award four $50 gift cards. The survey contained questions that comprehensively addressed general anxiety related to finances using the validated Financial Anxiety Scale (FAS)^6,47^. This study was approved by The University of Texas MD Anderson Cancer Center Institutional Review Board and was done in accordance with the principles outlined in the Declaration of Helsinki.

### Participants

This study included adults (> 18 years) with a diagnosis of arthritis, cardiovascular disease, diabetes, HIV/AIDS, multiple sclerosis, chronic obstructive pulmonary disease, asthma, autoimmune disease, inflammatory bowel disease, hypertension, and cancer who had previously received social needs navigation or financial aid from PAF. PAF beneficiaries often demonstrate limited resources and financial challenges to cover out-of-pocket expenses, psychological distress, and adaptive coping mechanisms^46,48^. Participants had to have a valid email address, be at least 18 years old, have given their prior consent to receive survey communications from PAF, and be able to complete the survey in English to be included in the study.

### Outcome and Covariates

 Our primary aim is to investigate the independent relationship of demographic characteristics, socioeconomic factors, and an active cancer diagnosis with financial anxiety using the FAS. The FAS is a validated seven-item measure corresponding with symptoms of an anxious disposition towards one’s financial status^6,47^. Scores range from 7 to 49, and higher scores denote higher financial anxiety. Survey responses were linked to the following patient-level information abstracted from the PAF database: age, self-reported sex, race and ethnicity, marital status (i.e., single, married, divorced), annual household income, and household size. Information relating to current education level, employment status, insurance coverage (i.e., Medicare, Medicaid, Consolidated Omnibus Budget Reconciliation Act (COBRA), Private), and self-reported rural–urban-suburban status were collected directly within the survey tool. Comorbidities were classified as 0 (primary condition only), 1–2, or ≥ 3 number of comorbidities. Active cancer diagnosis was used to characterize patients who received PAF services pursuant of cancer treatment and treated as an independent variable. Our justification for this exploration is based on the well-established association between cancer and financial toxicity, relative to other chronic conditions^49,50^. Additionally, financial toxicity in the setting of cancer treatment has been shown to significantly dampen global- and condition-specific quality-of-life^51^.

### Statistical analysis

Continuous variables were summarized using descriptive statistics such as means, standard deviations (SD), medians, and interquartile ranges (IQR). Frequencies and percentages were used to present categorical variables. Multivariable linear regression models were used to assess the effect of demographic and socioeconomic characteristics on FAS using beta coefficients (β) and corresponding 95% confidence intervals (CI). Factors with type III *p* value < 0.1 were included in the regression model. All models were adjusted for age, sex, race and ethnicity, region, annual household income, household size, marital status, employment status, chronic condition type, and number of comorbidities. We utilized a SAS procedure (proc glmselect) and specified the Akaike selection criterion as our selection criteria for a stepwise model selection method to fit the most parsimonious statistical models. The fitting criteria can be found in the supplementary Table S1. Missing data were imputed using multivariate imputation by chained equations (MICE)^52^. Ten sets of imputed data were used in data analysis and the MIANALYZE procedure was employed to integrate the estimations^53^. The proportion of imputed FAS data was 9.14% (n = 162/1771). All statistical tests were 2-sided, and *p* values less < 0.05 were considered statistically significant. All analyses were performed in SAS Enterprise Guide version 9.4 (SAS Institute Inc., Cary, NC, USA).

### Informed consent

Informed consent was obtained from all participants included in the study prior to receiving survey communications from the Patient Advocate Foundation. This is an observational study, and The University of Texas MD Anderson Cancer Center issued expedited IRB approval.

## Supplementary Information

Supplementary Table S1.

## Data Availability

The datasets generated during and/or analyzed during the current study are not publicly available due to institutional policies but are available from the corresponding author on reasonable request.
