# Supplementary material for: A nationwide cross-sectional study on the association of patient-level factors with financial anxiety in the context of chronic medical conditions
Source: Sci Rep. 2023 Jun 26;13:10363. doi: 10.1038/s41598-023-36282-2 (PMC10293194; doi:10.1038/s41598-023-36282-2)
Supplement: Supplementary file 1 — Supplementary Table S1. [file 41598_2023_36282_MOESM1_ESM.docx]

**Supplementary Table S1**. Fitting criteria for the multivariable regression models

| **Criteria For Assessing Goodness Of Fit** | | | |
| --- | --- | --- | --- |
| **Criterion** | **DF** | **Value** | **Value/DF** |
| **Deviance** | 1579 | 198802.0902 | 125.9038 |
| **Scaled Deviance** | 1579 | 1609.0000 | 1.0190 |
| **Pearson Chi-Square** | 1579 | 198802.0902 | 125.9038 |
| **Scaled Pearson X2** | 1579 | 1609.0000 | 1.0190 |
| **Log Likelihood** |  | -6158.1048 |  |
| **Full Log Likelihood** |  | -6158.1048 |  |
| **AIC** |  | 12378.2096 |  |
| **AICC** |  | 12379.4677 |  |
| **BIC** |  | 12545.0940 |  |
